# Trajectories of childhood adversity, social welfare dependence in young adulthood, and the mediating role of mental health problems: a Danish population‐based cohort study

**DOI:** 10.1111/jcpp.70062

**Published:** 2025-10-03

**Authors:** Tjeerd Rudmer de Vries, Signe Kær Bennetsen, Leonie K. Elsenburg, Signe Hald Andersen, Bertina Kreshpaj, Karsten Thielen, Naja Hulvej Rod

**Affiliations:** ^1^ Department of Public Health, Copenhagen Health Complexity Center University of Copenhagen Copenhagen Denmark; ^2^ ROCKWOOL Foundation Interventions Unit Copenhagen Denmark; ^3^ Department of Occupational and Social Medicine University Hospital of Holbæk Holbæk Denmark

**Keywords:** Mental health, social welfare, childhood adversity, mediation, young adults

## Abstract

**Background:**

Childhood adversity is associated with increased risks of long‐term social welfare dependence in young adulthood. Mental health problems may mediate this relation, but evidence remains lacking.

**Methods:**

613,643 individuals from the Danish Life Course cohort (DANLIFE) were categorized into five trajectory groups based on their annual exposure to adversity: low adversity, early‐life material deprivation, persistent material deprivation, loss or threat of loss, or high adversity. Mental health problems were identified through hospital contacts and psychotropic medication use. Long‐term social welfare dependence was defined as receiving social benefits for at least 52 consecutive weeks within the follow‐up period. We examined the contribution of differential exposure and susceptibility to mental health problems in relation to childhood adversity and long‐term social welfare dependence through causal mediation analysis.

**Results:**

The different childhood adversity groups saw 54–319 additional cases of long‐term social welfare dependence per 1,000 individuals compared with the low adversity group. These associations were partly mediated through mental health problems. To illustrate, in the high adversity group, differential exposure to mental health problems accounted for 15.0% (95% CI: 14.4–15.6) of the total effect, while differential susceptibility accounted for an additional 9.8% (95% CI: 8.8–10.9).

**Conclusions:**

Mental health problems partly mediate the relation between childhood adversity and long‐term social welfare dependence in young adulthood through both elevated exposure and increased susceptibility. Addressing mental health problems and increasing resilience among individuals with a history of childhood adversity may mitigate the risk of subsequent social welfare dependence.

## Introduction

Childhood adversities such as poverty, parental separation, and parental somatic or mental illness are associated with social welfare dependence in early adulthood (de Vries, Arends, Oldehinkel, & Bültmann, 2023; Kreshpaj et al., 2024; Lund, Andersen, Winding, Biering, & Labriola, 2013). Long‐term social welfare dependence in young adulthood can be highly problematic for individuals and constitutes an important societal challenge. Studies have shown that individuals dependent on social welfare benefits are at increased risk of marginalization, social exclusion, and long‐term health issues (Luijkx & Wolbers, 2009; Shahidi, Ramraj, Sod‐Erdene, Hildebrand, & Siddiqi, 2019). Moreover, social welfare dependence incurs significant costs at a societal level, especially in countries with extensive social security systems like Denmark (DST, 2023). These societal costs are further compounded by the broader consequences of childhood adversities and mental health problems, both at a societal and individual level (Hughes et al., 2017, 2021). Although the association between childhood adversity and social welfare recipience is well documented, the pathways underlying this association remain under‐investigated.

Mental health problems have been suggested to play an important role in explaining the association between childhood adversity and social welfare dependence in young adulthood (de Vries et al., 2023). Young adulthood is suggested to be a stressful period in life, not least because of several important transitions that young adults undergo during this period (e.g., the school‐to‐work transition) (Bültmann et al., 2020; Settersten, 2003). Several mental health problems have a peak age of onset during this period (e.g., substance use disorders, mood disorders), and the global onset of the first mental disorder occurs before age 25 in 62.5% of individuals (Solmi et al., 2022). Through a variety of developmental changes (e.g., increased stress‐reactivity, changes in emotional processing), individuals with a history of childhood adversity, and especially those that relate to household dysfunction (e.g., parental addiction) or maltreatment, are particularly likely to experience mental health problems during young adulthood (Baldwin et al., 2022; de Vries et al., 2023; Ellis, Sheridan, Belsky, & McLaughlin, 2022; McCrory & Viding, 2015; Pollmann, Fritz, Barker, & Fuhrmann, 2023). Experiencing mental health problems during this period of life may negatively affect individuals as they traverse the school‐to‐work transition (Bültmann et al., 2020), increasing the likelihood that they become dependent on social welfare benefits as they grow older (Merikukka, Ristikari, Tuulio‐Henriksson, Gissler, & Laaksonen, 2018; OECD, 2021; Weavers et al., 2021). Mental health problems represent a potentially highly relevant, and malleable, intervention target to reduce the risk of social welfare dependence following exposure to childhood adversity.

Although a few studies have provided preliminary evidence for the mediating role of mental health problems in the association between childhood adversity and social welfare dependence in young adulthood (Henry, Celia, & Merrick, 2018; Jaffee et al., 2018; 2024), several knowledge gaps yet remain. Previous studies focused on childhood maltreatment only, largely ignoring other, more common childhood adversities (e.g., parental separation) that are known to be associated with both mental health problems and social welfare dependence (Baldwin et al., 2022; de Vries et al., 2023; Lund et al., 2013). Moreover, previous studies have predominantly focused on whether individuals with a history of childhood adversity are differentially exposed to mental health problems (Henry et al., 2018; Jaffee et al., 2018), thus ignoring the possibility that part of the mediating effect of mental health problems may be due to differential susceptibility (Diderichsen, Hallqvist, & Whitehead, 2019).

Differential susceptibility in this context reflects that individuals with a history of childhood adversity are differentially affected by mental health problems during the school‐to‐work transition than individuals without a history of childhood adversity. Previous research suggests that individuals with a history of childhood adversity may be less resilient to the impact of mental health problems because they typically have lower educational attainment and limited social support structures, making them more likely to become reliant on social benefits when facing mental health problems (Bussemakers & Kraaykamp, 2020; Jaffee et al., 2018). Previous research highlights the potential importance of differential susceptibility to mental health problems in individuals with a history of childhood abuse in relation to labour market inactivity, but sample size limitations render this evidence inconclusive (de Vries, Arends, Oldehinkel, & Bültmann, 2024). Additional evidence for the importance of mental health problems in the relationship between childhood adversity and social welfare dependence is thus highly warranted.

**Figure 1:**
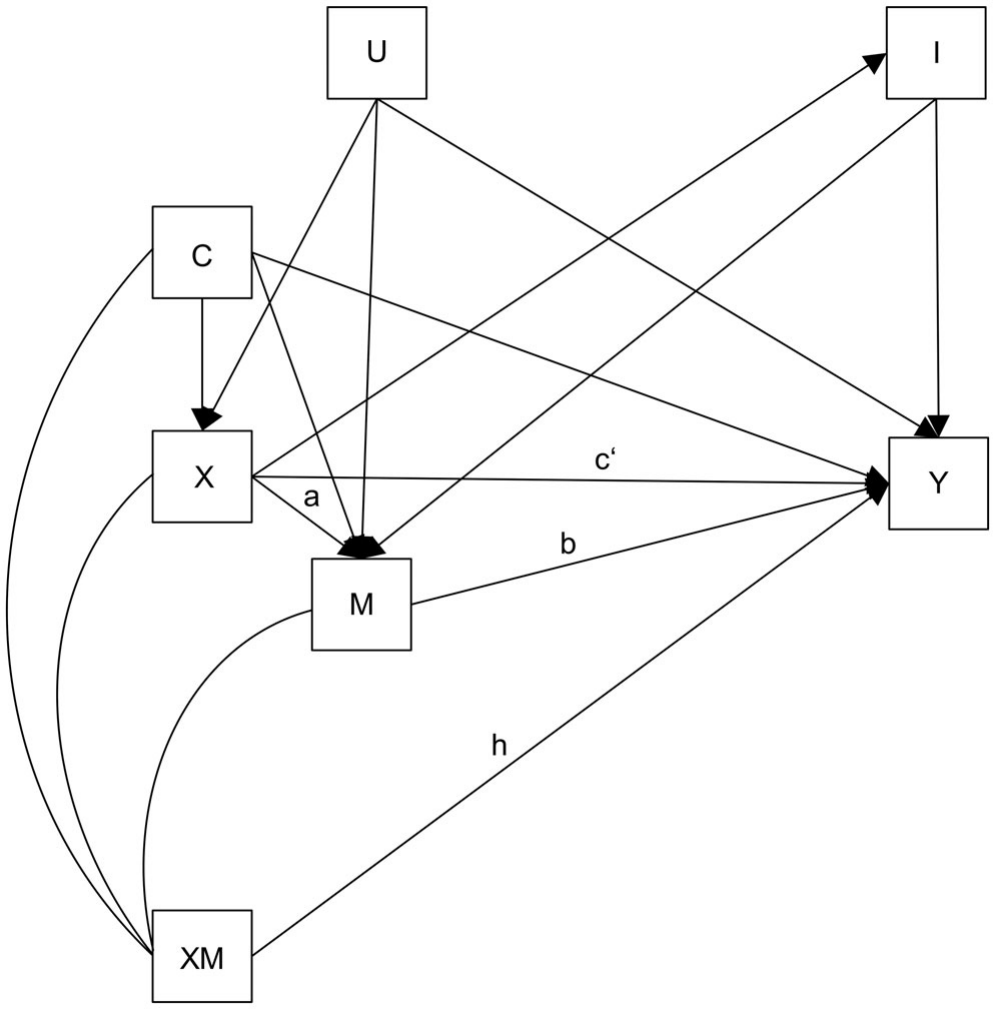
Path diagram. Path diagram depicting the links between trajectories of childhood adversity (X), mental health problems (M), baseline covariates (C), unmeasured confounders (U), unmeasured intermediate confounders (I), and the interaction between trajectories of childhood adversity and mental health problems (XM). Paths a and b reflect differential exposure, path h reflects differential susceptibility, and path c' reflects the direct effect of childhood adversity on long‐term social welfare dependence not through mental problems

In this study, we aimed to investigate and decompose the role of mental health problems between ages 16 and 24 in the association of childhood adversity between ages 0–15 and long‐term social welfare dependence between ages 25 and 30 (see Figure 1). We hypothesized that mental health problems mediate the relation between childhood adversity and long‐term social welfare dependence. We further hypothesized that this mediating effect can be explained by both differential exposure and differential susceptibility.

## Methods

### Participants

**Figure 2:**
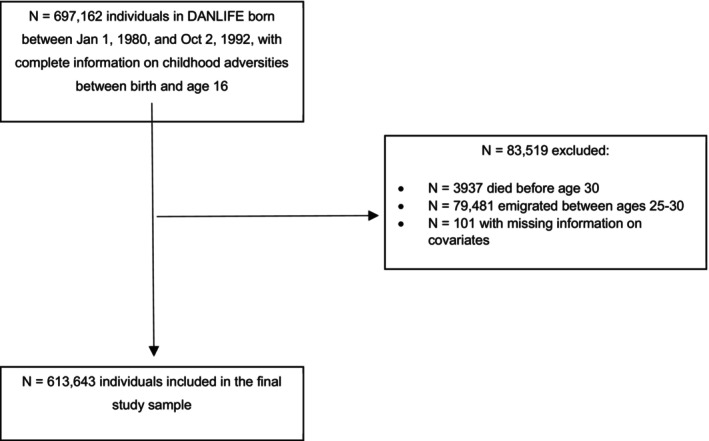
Study flowchart

This study used data from the register‐based Danish Life Course (DANLIFE) cohort, which includes children born in Denmark since 1980 (Bengtsson, Dich, Rieckmann, & Hulvej Rod, 2019). The sample used for this study is restricted to individuals born between 1 Jan 1980, and 2 Oct 1992, with complete information on childhood adversity to ensure follow‐up until age 30 (*N* = 697,162). From this sample, we excluded individuals who passed away (*N* = 3,937) or emigrated during follow‐up (*N* = 79,481). After excluding individuals with missing information on included covariates (*N* = 101), the final study sample consisted of 613,643 individuals (see Figure 2).

### Measures

#### Trajectories of childhood adversity between ages 0 and 15

**Figure 3:**
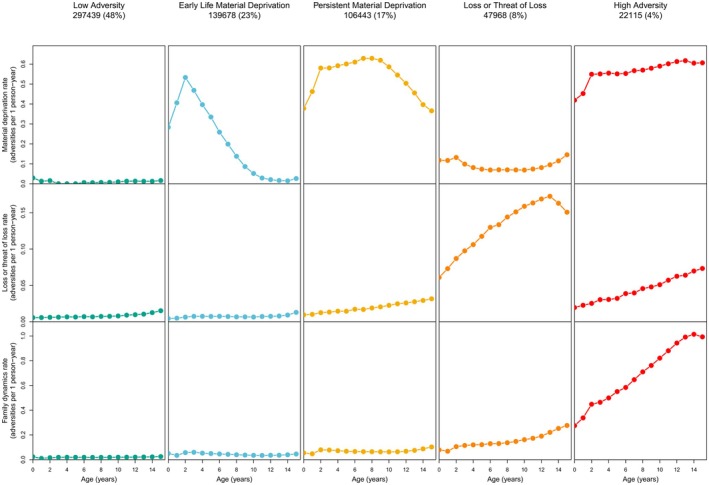
Visual description of the childhood adversity trajectory groups

DANLIFE includes 12 different childhood adversities, divided into three dimensions: material deprivation (family poverty and parental long‐term unemployment), loss or threat of loss (parental somatic illness, sibling somatic illness, parental death, sibling death), and family dynamics (foster care placement, parental psychiatric illness, sibling psychiatric illness, parental alcohol abuse, parental drug abuse, maternal separation) (Bengtsson et al., 2019; Rod et al., 2020). Through group‐based multi‐trajectory modelling, the children were previously classified in one of five childhood adversity trajectory groups based on the count of the child's annual exposure to adversity in each of the three dimensions between 0 and 15 years (Rod et al., 2020): low adversity, early‐life material deprivation, persistent material deprivation, loss or threat of loss, or high adversity (Figure 3).

We refer the reader to Appendix S1 for more information about the underlying trajectories, including average posterior probabilities in the original and full DANLIFE cohort.

#### Mental health problems between ages 16 and 24

Information on mental health problems was based on diagnoses through inpatient or outpatient contacts in public and private hospitals as recorded in the Danish Psychiatric Central Research Register, and psychotropic medication prescriptions by physicians and picked up at pharmacies in Denmark as recorded in the National Prescription Registry (Mors, Perto, & Mortensen, 2011; Pottegård et al., 2016). Diagnoses included a variety of mental and behavioural disorders from the International Statistical Classification of Diseases and Related Health Problems, Tenth Revision (ICD‐10), that commonly arise during adolescence and young adulthood (Solmi et al., 2022) and are typically transient in nature. We excluded neurodevelopmental conditions (e.g., autism spectrum disorder, attention‐deficit hyperactivity disorder) due to their non‐transient nature and complex interactions with childhood adversity (Gajwani & Minnis, 2023). Diagnoses were supplemented with prescriptions because the hospital registers contain no record of mental health problems diagnosed outside the hospital, including those diagnosed by general practitioners and private psychiatrists. Two prescriptions or more were classified as mental health problems as one prescription may be more indicative of transient use. Information on included diagnoses (ICD‐10 codes) and psychotropic medications (ATC groups), and descriptive statistics of their distribution across the trajectories of childhood adversity can be found in Appendix S2 and Table S1.

#### Social welfare dependence between ages 25 and 30

Information on social welfare dependence was extracted from the Danish Register for Evaluation of Marginalization (DREAM), which is based on information from the Danish Ministry of Employment, Ministry of Education, the Danish Civil Registration System, and the Danish Tax Agency (Hjollund, Larsen, & Andersen, 2007). DREAM provides information on weekly use of social benefits across Denmark and covers all individuals who received social benefits and other forms of transfer income from July 1991 onwards. We assessed 14 social benefit categories including social assistance, unemployment support, and early retirement pension, all of which are further described in Table S2. Social welfare dependence was operationalized as having received any of the included benefits for at least 52 consecutive weeks within the follow‐up period, which corresponds to the period between the week of individuals' 25th birthday and the week of their 30th birthday. Descriptive statistics of the distribution of social benefit categories among long‐term users across the trajectories of childhood adversity can also be found in Tables S2 and S3.

#### Co‐variates

Following previous literature, we adjusted for sex assigned at birth (male/female), birth year, parental country of origin (Western/non‐Western), and maternal age at time of birth (<20 years/20–30 years/>30 years) to address confounding (Bager, Laursen, Palic, Nordin, & Høgh Thøgersen, 2022; Elsenburg et al., 2022; Haahr‐Pedersen et al., 2020; McLaughlin, Costello, Leblanc, Sampson, & Kessler, 2012; Walsh, McCartney, Smith, & Armour, 2019). Parental country of origin was defined as Western if one or both parents were of European, North American, Australian, or New Zealand descent and non‐Western if both parents were from elsewhere.

### Statistical analysis

We estimated the mediating effect of mental health problems in the association between trajectories of childhood adversity and social welfare dependence through counterfactual mediation analysis with the three‐way decomposition approach (Lange, Vansteelandt, & Bekaert, 2012). The three‐way decomposition approach allowed us to decompose the total effect of childhood adversity on social welfare dependence into three components: a component due to differential exposure to mental health problems across the childhood adversity groups (mediation only), a component due to differential susceptibility to mental health problems (mediated interaction), and a component reflecting the effect of childhood adversity on social welfare dependence that is not due to mental health problems (the direct effect). Table 1 contains an overview of how these three components can be interpreted in the context of our study.

**Table 1:** Explanation of the three‐way decomposition through which trajectories of childhood adversity affect long‐term social welfare dependence in young adulthood

Differential exposure	The effect of childhood adversity on long‐term social welfare dependence is due to individuals with a history of childhood adversity experiencing higher levels of mental health problems than those without such a history
Differential susceptibility	The effect of mental health problems on social welfare dependence is stronger among those with a history of childhood adversity than those without such a history
Direct effect	The effect of childhood adversity on social welfare dependence that is not due to differences in exposure or susceptibility to mental health problems

To obtain our estimates of interest, we applied the natural effects model combined with the imputation approach as implemented in the R package medflex (version 0.6.7). The natural effects model directly parametrizes the target causal estimands on their most natural scale, facilitating formal testing and interpretation of the obtained estimates as compared to other approaches (Lange et al., 2012). The analytical pipeline of this approach consists of two steps. First, the data are expanded to estimate the risk of the outcome under all possible versions of the exposure (factual and counterfactual exposure to the childhood adversity groups) while keeping the mediator and covariates constant. Second, we fitted a binomial regression model with the identity link function to the expanded data to obtain our estimates of interest using the low adversity group as the reference group. Estimates are reported as risk differences per 1,000 individuals and their corresponding 95% confidence intervals for both the total sample and for men and women separately. All binomial regression analyses included an interaction term between childhood adversity and mental health problems to obtain estimates for differential susceptibility to mental health problems across the adversity groups.

We furthermore calculated proportions of the total effect due to the three decomposed effects and estimated the associated 95% confidence intervals through simulation in the MASS package (version 7.3‐60.0.1). The 95% confidence intervals were obtained by creating distributions of proportions across 10^6^ simulations for each of the decomposed effects and taking the range between the 2.5th and the 97.5th percentiles of these distributions.

#### Sensitivity analyses

We conducted several sensitivity analyses to assess the robustness of our findings. We first estimated two additional models in which we first adjusted for parental educational background (low [<10 years], middle [10–12 years], or high [>12 years]), and subsequently also for small for gestational age (birth weight below the 10th percentile of age‐ and sex‐specific reference curves for intrauterine growth) and pre‐term birth (before 37 weeks of pregnancy) in a sample with full information on these factors (*N* = 592,291). These three factors have been suggested to confound the associations between childhood adversity, mental health problems, and social welfare dependence (Bilsteen, Taylor‐Robinson, Børch, Strandberg‐Larsen, & Nybo Andersen, 2018; Haula & Vaalavuo, 2021; Holstein et al., 2021; Moster, Lie, & Markestad, 2008). At the same time, parental educational background is likely to be highly correlated with, especially the material deprivation dimension of the childhood adversity measure (Elsenburg et al., 2022), and small for gestational age and pre‐term birth may potentially lie on the causal pathway (as adversity may already be present during pregnancy) instead of being confounders (Dejin‐Karlsson et al., 2000; Voit et al., 2022). As these factors could both be confounding or mediating our effects of interest, we only adjusted for them in a sensitivity analysis. Finally, we restricted the analyses to include only mental health diagnoses to test the robustness of our findings.

## Results

Of the 613,643 individuals included in this study, approximately half were women (48.2%) and 98.7% of individuals had parents of Western origin. Maternal age at birth was between 20 and 30 years of age for most individuals. Table 2 provides an overview of these descriptive statistics for the total sample as well as across the five childhood adversity groups.

**Table 2:** Descriptive statistics of the study population by childhood adversity trajectory group membership and overall

Variable	Low adversity	Early‐life material deprivation	Persistent material deprivation	Loss or threat of loss	High adversity	Overall
	*N* = 297,439	*N* = 139,678	*N* = 106,443	*N* = 47,968	*N* = 22,115	*N* = 613,643
Sex assigned at birth						
Female	143,297 (48.2%)	67,585 (48.4%)	51,669 (48.5%)	23,349 (48.7%)	10,062 (45.5%)	295,962 (48.2%)
Male	154,142 (51.8%)	72,093 (51.6%)	54,774 (51.5%)	24,619 (51.3%)	12,053 (54.5%)	317,681 (51.8%)
Origin of parents						
Non‐Western	1,228 (0.4%)	1,685 (1.2%)	4,389 (4.1%)	689 (1.4%)	172 (0.8%)	8,163 (1.3%)
Western	296,211 (99.6%)	137,993 (98.8%)	102,054 (95.9%)	47,279 (98.6%)	21,943 (99.2%)	605,480 (98.7%)
Maternal age at birth						
<20 years	3,136 (1.1%)	5,602 (4%)	8,528 (8.0%)	1,931 (4.0%)	2,659 (12.0%)	21,856 (3.6%)
20–30 years	210,859 (70.9%)	108,527 (77.7%)	78,466 (73.7%)	32,846 (68.5%)	15,623 (70.6%)	446,321 (72.7%)
>30 years	83,444 (28.1%)	25,549 (18.3%)	19,449 (18.3%)	13,191 (27.5%)	3,833 (17.3%)	145,466 (23.7%)
Year of birth						
1980–1983	85,176 (28.6%)	33,209 (23.8%)	39,216 (36.8%)	12,879 (26.8%)	5,908 (26.7%)	176.388 (28.7%)
1984–1987	87,042 (29.3%)	40,122 (28.7%)	33,966 (31.2%)	13,673 (28.5%)	6,736 (30.5%)	181,539 (29.6%)
1988–1992	125,221 (42.1%)	66,347 (47.5%)	33,261 (31.2%)	21,416 (44.6%)	9,471 (42.8%)	255,716 (41.7%)
Mental health problems	42,847 (14.4%)	26,798 (19.2%)	22,642 (21.3%)	11,849 (24.7%)	8,806 (39.8%)	112,942 (18.4%)
Social welfare dependence	28,160 (9.5%)	21,351 (15.3%)	23,820 (22.4%)	9,642 (20.1%)	9,368 (42.4%)	92,341 (15.0%)

### Childhood adversity, mental health problems and long‐term welfare dependence

**Figure 4:**
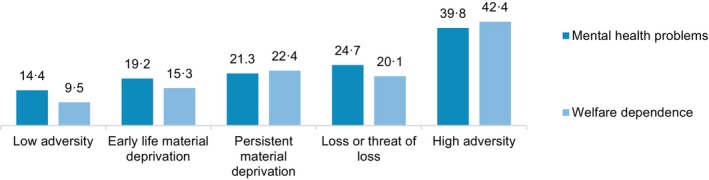
Percentage of individuals with mental health problems (ages 16–24) and long‐term social welfare dependence (ages 25–30) across the five childhood adversity groups

Both mental health problems (18.4%) and long‐term social welfare dependence (15.0%) were experienced by approximately one‐sixth of the sample. Figure 4 shows that mental health problems and social welfare dependence were unevenly distributed across the five adversity groups.

Table 3 shows the total effect of childhood adversity on social welfare dependency and the decomposition of the total effect into the direct effect and differential exposure and susceptibility to mental health problems. Compared with individuals in the low adversity group, individuals from all other adversity groups were more likely to be dependent on social welfare benefits for 52 consecutive weeks between the ages of 25 and 30. Risk differences ranged from 54 (95% CI: 52–56) additional cases of welfare dependence per 1,000 individuals in the early material deprivation group to 319 (95% CI: 312–325) additional cases of welfare dependence per 1,000 individuals in the high adversity group.

**Table 3:** Total effect of childhood adversity on social welfare dependence and three‐way decomposition

Low adversity	Total effect	Differential exposure		Differential susceptibility		Direct effect	
	Additional cases per 1,000 individuals	Additional cases per 1,000 individuals	% of total effect	Additional cases per 1,000 individuals	% of total effect	Additional cases per 1,000 individuals	% of total effect
	Ref.	Ref.	Ref.	Ref.	Ref.	Ref.	Ref.
Early material deprivation	54 (52–56)	7 (7–8)	13.7 (12.7–14.7)	3 (3–3)	5.5 (4.9–6.2)	43 (41–45)	80.8 (79.6–82.0)
Persistent material deprivation	121 (119–124)	13 (12–13)	10.5 (9.9–11.0)	9 (9–10)	7.8 (7.3–8.3)	99 (97–102)	81.7 (81.0–82.4)
Loss or threat of loss	103 (99–106)	19 (18–20)	18.5 (17.5–19.5)	9 (8–10)	8.9 (7.9–9.9)	75 (71–78)	72.6 (71.2–74.0)
High adversity	319 (312–325)	48 (46–50)	15.0 (14.4–15.6)	31 (28–35)	9.8 (8.8–10.9)	240 (233–246)	75.2 (74.0–76.3)

Discrepancies in summation to total effects are due to rounding. Estimates are adjusted for sex, birth year, parental country of origin, and maternal age at the time of birth. The estimates for differential exposure, differential susceptibility, and the direct effect sum up to the total effect of each of the childhood adversity trajectories on social welfare dependence.

### The mediating effect of mental health problems

The total indirect effect (sum of differential exposure and susceptibility) through mental health problems across the different childhood adversity groups ranged from 10 (95% CI: 10–11) additional cases of welfare dependence per 1,000 individuals in the early material deprivation group to 79 (95% CI: 75–83) additional cases of welfare dependence per 1,000 individuals in the high adversity group. The percentages of the total effect accounted for by the total indirect effect ranged between 18.3% (95% CI: 17.6–19.0) in the persistent material deprivation group and 27.4% (95% CI: 26.0–28.8) in the loss or threat of loss group. These findings confirm our hypothesis that mental health problems mediate the relation between childhood adversity and long‐term social welfare dependence.

### Differential exposure and susceptibility to mental health problems

In line with our hypothesis, we find that both differential exposure and susceptibility explain the mediating effect of mental health problems. Differential exposure to mental health problems accounted for 13.7% (95% CI: 12.7–14.7), 10.5% (95% CI: 9.9–11.0), 18.5% (95% CI: 17.5–19.5), and 15.0% (95% CI: 14.4–15.6) of the total effect for the early material deprivation, persistent material deprivation, loss or threat of loss, and high adversity group, respectively. Differential susceptibility to mental health problems accounted for 5.5% (95% CI: 4.9–4.3), 7.8% (95% CI: 7.3–8.3), 8.9% (95% CI: 7.9–9.9), and 9.8% (95% CI: 8.8–10.9) of the total effect across the childhood adversity groups, respectively. Sex‐stratified analyses indicated that estimates largely did not differ between men and women (Tables S4 and S5).

### Sensitivity analyses

In the sensitivity analysis in which we adjusted for parental educational background, the total effect of childhood adversity on social welfare dependence was attenuated across all childhood adversity groups, as expected. Both the absolute number of cases and proportion of the total effect due to differential exposure to mental health problems were attenuated, while the effects due to differential susceptibility increased across all groups in both absolute numbers and relative importance (Table S6). Adding small for gestational age and pre‐term birth did not further attenuate these estimates (results not shown). In the sensitivity analyses where we only used diagnoses for mental health problems, we found similar patterns as in the main analysis, although estimates for differential exposure and susceptibility were slightly weaker across all groups, as expected due to the cruder measure of mental health problems (Table S7).

## Discussion

In an unselected sample of 613,643 Danish individuals, we found clear evidence for a mediating role of mental health problems in the association between childhood adversity and social welfare dependence in young adulthood. Our findings support our hypothesis that the mediating effect of mental health problems is driven by mechanisms of both differential exposure and susceptibility, which have important ramifications for intervention strategies.

The results of this study confirm and extend previous findings that highlighted the importance of mental health problems in the association between childhood adversity and social welfare dependence (Henry et al., 2018; Jaffee et al., 2018; 2024). Where previous studies focused on childhood maltreatment only, our findings suggest that mental health problems also play a role for individuals who are exposed to a variety of other childhood adversities. The effects of mental health problems were most pronounced among individuals in the loss or threat of loss and high adversity groups. Interestingly, the mediating effects of mental health problems were weaker than those reported in previous studies. We expect that part of this discrepancy is due to the fact that we investigated a different set of adversities that may differ in severity and thus impact mental health problems (Baldwin et al., 2022). However, another potential reason for this discrepancy is that we relied on register‐based reports of both childhood adversities and mental health problems, whereas previous studies relied largely on (maternal) self‐report. Studies relying on self‐report typically show inflated associations between childhood adversities and mental health problems, likely due to reverse causation (individuals with mental health problems are more likely to perceive and report events as adverse) (Francis, Tsaligopoulou, Stock, Pingault, & Baldwin, 2023).

Our findings differ from previous studies in that we found that the mediating effect of mental health problems is not merely due to mechanisms of differential exposure, but also to mechanisms of differential susceptibility (de Vries et al., 2024). While we cannot provide insight into the mechanisms underlying differential susceptibility across the childhood adversity groups, it is likely that they differ across groups. For example, individuals in the material deprivation groups grew up in resource‐deprived families (e.g., poverty, long‐term unemployment), which are associated with lower educational attainment (Bussemakers & Kraaykamp, 2020). While similar mechanisms may be at play among individuals in the loss or threat of loss and high adversity groups, these individuals may also have weaker social support networks, for example due to an absence of contact with parents (Kong & Martire, 2019). Future research should further disentangle the mechanisms underlying differential susceptibility to mental health problems in relation to long‐term social welfare dependence in individuals exposed to childhood adversities.

While mental health problems play an important role in the association between childhood adversity and social welfare dependence, a large proportion of the association between childhood adversity and social welfare dependence is likely to be mediated through other pathways. Previous evidence suggests that educational attainment is particularly likely to play an important role (Bennetsen et al., 2025; Veldman, Bültmann, Almansa, & Reijneveld, 2015). As mentioned previously, however, educational attainment is also very likely to explain why some young adults are at an even higher risk of long‐term social welfare dependence than others (differential susceptibility). Similarly, other factors that may place the young adult in a more precarious position, for example through early parenthood, may also both mediate and increase young adults' vulnerability to mental health problems. Ultimately, these various factors – mental health problems, educational attainment, other important transitions during young adulthood such as parenthood, and physical health problems ‐ underlying the association between childhood adversity and social welfare dependence are likely to be bidirectionally associated and interacting over time together with social welfare dependence. Unravelling these complex dynamics, for example through network analytical approaches or systems dynamics models, should be a priority of future studies and may provide further insight as to the prioritization of intervention targets.

### Strengths and limitations

This study has several strengths. The large, unselected sample of Danish individuals with full follow‐up provided the statistical power required to distinguish between differential exposure and susceptibility to mental health problems in the association between childhood adversity and long‐term social welfare dependence. Our comprehensive measure of childhood adversity covering information on the co‐occurrence, timing, and duration of various childhood adversities is unique, and this level of detail is seldom obtained in survey‐based studies. Moreover, we included data on weekly use of a variety of social welfare benefits, which allowed us to obtain a broad understanding of social welfare dependence in young adults with a history of childhood adversity and mental health problems.

We also acknowledge several limitations. First, despite our comprehensive approach regarding the operationalization of childhood adversity, we were unable to include direct measures of childhood maltreatment because such information is not readily available in the Danish registers. Childhood maltreatment is likely to have occurred in a section of the high‐adversity group, especially, and may partially drive the association with social welfare dependence in this group. We did, however, include measures of placement in foster care, which capture the more severe and apparent cases of child maltreatment. More generally, while we believe that our operationalisation of childhood adversity captures broader adversity patterns in our population, we may have overlooked potential nuances between different vulnerable subgroups – for example, those with and without a history of maltreatment – that may require more precisely targeted support and resource allocation.

Second, while this study highlights the importance of mental health problems in the association between childhood adversity and social welfare dependence, we likely underestimated the true effect of mental health problems due to misclassification problems. The nature of register‐based operationalization of mental health problems means that individuals who are identified as having mental health problems have received some form of treatment, potentially attenuating our estimates under the assumption that treatment may have facilitated better outcomes among those receiving treatment. In addition, our operationalisation of mental health problems is likely to be subject to differential misclassification given that individuals with higher rates of childhood adversity are generally less likely to seek and receive help for their mental health problems than non or less‐affected peers (Koball et al., 2019, 2021). As a result, our study may have missed a group of individuals with hidden unmet needs that may require support. Indeed, previous research has shown that there are substantial levels of unmet care needs among young people both with and without diagnosed mental health problems (Jörg et al., 2016). It is thus likely that mental health problems contribute more substantially to the pathway between childhood adversity and social welfare dependence than our findings suggest, and that the present estimates may understate their full mediating role. To determine the impact of differential misclassification on the decomposition of the mediating effect specifically, future research may examine the impact of different misclassification scenarios to examine to what extent estimates of differential exposure and susceptibility differ across these scenarios.

Finally, causal mediation analysis relies on assumptions of identification (non‐confounding and no intermediate confounding) for causal interpretation of direct and indirect effects. Moreover, the identification assumptions implicitly require an assumption of temporal ordering (Valeri & VanderWeele, 2013). Residual confounding in our study may have arisen due to measurement error in adjusted covariates (Greenland, 1980) as well as unmeasured covariates, such as genetic influences (Baldwin et al., 2022). While studies have shown that a variety of childhood adversities (e.g., parental mental health problems) affect mental health problems after adjusting for such influences (Baldwin et al., 2022), future studies may utilize genetically informed samples to shed light on to what extent genetic influences explain the mediating role of mental health problems on long‐term social welfare dependence following childhood adversity. It is likely that factors such as educational attainment and social support confound the mediator–outcome relation in our study (Bussemakers & Kraaykamp, 2020; Jaffee et al., 2018). However, because evidence suggests that these factors are directly influenced by childhood adversity, they are considered intermediate confounders that would hamper the identification of the estimands of interest in this study. Combining all these factors simultaneously is an important avenue for future research.

### Implications

Our findings further highlight the importance of mental health problems in the association between childhood adversity and social welfare dependence, suggesting that mental health problems in adolescence and emerging young adulthood represent a central leverage point for preventive and early care interventions to potentially reduce later dependency on social welfare benefits. Given that both differential exposure and differential susceptibility play a role, interventions ought to be aimed at both reducing the occurrence of serious mental health problems, including early identification and treatment in vulnerable groups, as well as promoting resilience to mental health problems among individuals with a history of childhood adversity through, for example, external support networks (e.g., mentor systems) (Ahrens, DuBois, Richardson, Fan, & Lozano, 2008). Given that all mental health problems identified in this study indicate contact with healthcare professionals, we deem it relevant to highlight that these contacts represent a window of opportunity during which vulnerable young adults can be provided with tailored care to prevent further accumulation of disadvantages. Future studies on the mediating role of mental health problems in the association between childhood adversity and long‐term social welfare dependence may seek to further disentangle the severity of mental health problems, for example, by leveraging both survey and register data simultaneously. Furthermore, future studies could build on our findings by examining which support services, e.g., psychotherapy, vocational training, or peer support programs, could reduce mental health problems to avoid subsequent social welfare dependency among individuals with a history of childhood adversity. To further tailor these intervention and prevention efforts, such studies could also examine which types of support help which people at which ages and examine concomitant reductions in social welfare dependency.

While intervening on mental health problems may alleviate risks of long‐term social welfare dependence in young adulthood, the preferred strategy is to continue efforts to prevent childhood adversity from occurring in the first place. Our comprehensive approach to the measurement of adversity sheds light on broader patterns of adversity that children facing mental health problems and long‐term social welfare dependence may have experienced. While we are unable to pinpoint which exact childhood adversities ought to be focused on in policy and intervention, we have been able to identify specific populations at risk. Given that individuals most at risk of mental health problems and long‐term social welfare dependence experience multiple adversities across dimensions, constituents in policy and practice may use several adversities as leverage points to disrupt the occurrence of adversity and potential downstream consequences. To gain further insight into the role of specific adversities within the context of these broader patterns of adversity requires further conceptual and methodological innovation within the field of childhood adversity. For example, methods from environmental epidemiology such as kernel‐based regression approaches (Bobb et al., 2015) may be well suited to address this issue.

## Conclusion

Mental health problems play an important role in explaining the association between childhood adversity and social welfare dependence in young adulthood. Improving mental health and youth resilience to mental health problems represents highly relevant targets for intervention that may aid in disrupting the chain of risk between childhood adversity and social welfare dependence in the transition period into young adulthood. The healthcare system is an important setting in which vulnerable young adults with a history of childhood adversity and ongoing mental health problems may be identified, and where measures to prevent subsequent social welfare dependence may be initiated.

## Ethical information

Statistics Denmark and the Danish Health Data Authorities granted access to pseudonymized data from the Danish administrative and health registers. DANLIFE has been approved by the Danish Data Protection Agency through the records of research projects that involve personal data at the Faculty of Health and Medical Sciences, University of Copenhagen (Copenhagen, Denmark) (record no 514‐0641/21‐3000). According to Danish law, registry linkage studies do not require informed consent or ethical approval by the Danish National Committee on Health Research Ethics.

**Key points**

**What's known?**

Childhood adversity is strongly associated with social welfare dependence in young adulthood. Mental health problems in adolescence are suggested to play an important role in this association, but evidence remains scarce.

**What's new?**

Mental health problems explain the association between childhood adversity and social welfare dependence through mechanisms of both differential exposure and susceptibility.

**What's relevant?**

Intervening in mental health problems represents a valuable target for reducing social welfare dependence following exposure to various constellations of childhood adversity. Such interventions should focus on both reducing the occurrence of mental health problems as well as promoting resilience to mental health problems.

## Supporting information


**Appendix S1.** Group‐based multi‐trajectory model.
**Appendix S2.** Overview included diagnoses and prescriptions.
**Table S1.** Descriptive statistics of diagnoses and psychotropic medication usage across childhood adversity groups.
**Table S2.** Overview included benefit types.
**Table S3.** Descriptive statistics of social benefit usage among long‐term users across childhood adversity groups.
**Table S4.** Total effect of childhood adversity on social welfare dependence and three‐way decomposition, women only.
**Table S5.** Total effect of childhood adversity on social welfare dependence and three‐way decomposition, men only.
**Table S6.** Total effect of childhood adversity on social welfare dependence and three‐way decomposition, adjusted for parental education.
**Table S7.** Total effect of childhood adversity on social welfare dependence and three‐way decomposition, diagnoses only.

## Data Availability

It is not possible to share the individual participant data used for this study. Inquiries about secure access to the DANLIFE data under conditions stipulated by the Danish Data Protection Agency can be directed to the principal investigator of the study, Naja Hulvej Rod (nahuro@sund.ku.dk).
