# Supplementary material for: Trajectories of childhood adversity, social welfare dependence in young adulthood, and the mediating role of mental health problems: a Danish population‐based cohort study
Source: J Child Psychol Psychiatry. 2025 Oct 3;67(5):707–17. doi: 10.1111/jcpp.70062 (PMC13102053; doi:10.1111/jcpp.70062)
Supplement: Supplementary file 1 — Appendix S1. Group‐based multi‐trajectory model. Appendix S2. Overview included diagnoses and prescriptions. Table S1. Descriptive statistics of diagnoses and psychotropic medication usage across childhood adversity groups. Table S2. Overview included benefit types. Table S3. Descriptive statistics of social benefit usage among long‐term users across childhood adversity groups. Table S4. Total effect of childhood adversity on social welfare dependence and three‐way decomposition, women only. Table S5. Total effect of childhood adversity on social welfare dependence and three‐way decomposition, men only. Table S6. Total effect of childhood adversity on social welfare dependence and three‐way decomposition, adjusted for parental education. Table S7. Total effect of childhood adversity on social welfare dependence and three‐way decomposition, diagnoses only. [file JCPP-67-707-s001.docx]

**Trajectories of childhood adversity, social welfare dependence in young adulthood and the mediating role of mental health problems: a Danish population-based cohort study**

**Supporting Information**

**Appendix S1.** Group-based multi-trajectory model

The trajectories of childhood adversity were originally derived using a group-based multi-trajectory modelling (Nagin, Jones, Passos & Tremblay, 2018). The Stata package TRAJ was used to fit group-based multi-trajectory models using zero-inflated Poisson regressions that models the trajectories with a cubic function of age. The model yields a probability for each individual of being in each trajectory group. Average posterior probabilities in the original sample were 0.92 (SD = 0.18) for the low adversity group, 0.82 (SD = 0.17) for the early life material deprivation group, 0.89 (SD = 0.16) for the persistent material deprivation group, 0.83 (SD = 0.18) for the loss or threat of loss group, and 0.94 (SD = 0.13) for the high adversity group.

Due to computational issues with large data, a 2-stage approach was used: First, a model was fitted based on a random sample of 50,000 individuals. Second, the estimated probabilities of being in each trajectory group were then used to assign all individuals in the full cohort to their most likely trajectory group. To assess the consistency of estimating the model on a sub-sample, the procedure was performed on 5 random samples of 50000 individuals. Each of these returned five almost identical models.

**Appendix S2.** Overview included diagnoses and prescriptions

**ICD-10 codes:**

F10-F19.9 (Mental and behavioural disorders due to psychoactive substance use)
F20-F29 (Schizophrenia, Schizotypal, delusional, and other non-mood psychotic disorders)
F30-F31.9, F32-F34.0 and F38-F39 (Mood [Affective] disorders)
F40-F48.9 (Anxiety, dissociative, stress-related, somatoform and other non-psychotic mental disorders
F50-F51.9 (Behavioral syndromes associated with physiological disturbances and physical factors)
F60-F61 (Disorders of adult personality and behavior)

**Medication (ATC groups):**

N05A (Antipsychotics)

N0BA (Anxiolytics) except for N0BA (Benzodiazepine derivatives)
N06A (Antidepressants)
N05AN (Lithium)

N07BB (Alcohol dependence medication)

N07BC (Opiod dependence medication)

**Table S1.** Descriptive statistics of diagnoses and psychotropic medication usage across childhood adversity groups

| Variable | Low adversity  N = 297,439 | Early life material deprivation  N = 139,678 | Persistent material deprivation  N = 106,443 | Loss or threat of loss  N = 47,968 | High adversity  N = 22,115 |
| --- | --- | --- | --- | --- | --- |
| **Diagnoses** |  |  |  |  |  |
| F1 | 21073 | 15974 | 15738 | 8858 | 12026 |
| F2 | 17375 | 11265 | 11072 | 6613 | 7898 |
| F3 | 17907 | 11192 | 8283 | 5729 | 3480 |
| F4 | 26232 | 17690 | 15244 | 9744 | 7632 |
| F5 | 9391 | 4058 | 3057 | 2143 | 1050 |
| F6 | 15002 | 11320 | 10582 | 7083 | 7424 |
| **Medication** |  |  |  |  |  |
| N05A | 17489 | 11940 | 10603 | 6725 | 7204 |
| N05B | 1170 | 785 | 669 | 338 | 305 |
| N06A | 54436 | 34760 | 27787 | 15474 | 10024 |
| N07B | 1406 | 1242 | 1484 | 720 | 1110 |

**Table S2.** Overview included benefit types

| **DREAM codes** | **Benefit type (Danish name of benefit)** |
| --- | --- |
| 130-139, 730-739  111,213-219, 299  140, 143-149, 720, 723-729  151  152  153  700-709, 160-169  740-748  750-758  760-768  783  784  810-818  870-878  890, 893-899 | Social assistance (‘Kontanthjælp’)  Unemployment benefits (‘Dagpenge’)  Educational support (’Uddannelseshjælp’)  Special educational support (‘Særlig uddannelsesydelse’)  Labour market benefit (‘Arbejdsmarkedsydelse’)  Cash benefit (‘Kontantydelse’)  Integration benefit (‘Integrationsydelse’)  Unemployment support (‘Ledighedsydelse’)  Pre-rehabilitation benefit (‘For revalidering’)  Rehabilitation benefit (‘Revalidering’)  Early retirement pension (‘Førtidspension’)  Early retirement pension/Social assistance (‘Førtidspension/kontanthjælp’)  Resource course benefit (‘Ressourceforløbsydelse’)  Job assessment (‘Jobafklaring’)  Sickness benefit (‘Sygedagpenge’) |
| ***Note:*** *The names of some of the benefits were translated for the purpose of this study* | |

**Table S3.** Descriptive statistics of social benefit usage among long-term users across childhood adversity groups

| Variable | Low adversity  N = 297,439 | Early life material deprivation  N = 139,678 | Persistent material deprivation  N = 106,443 | Loss or threat of loss  N = 47,968 | High adversity  N = 22,115 |
| --- | --- | --- | --- | --- | --- |
| Social assistance | 26140 | 23353 | 37820 | 12222 | 14426 |
| Unemployment benefits | 27315 | 16618 | 16574 | 5082 | 2735 |
| Educational support | 14757 | 16575 | 16727 | 7828 | 8935 |
| Special educational support | 181 | 173 | 195 | 49 | 35 |
| Labour market benefit | 350 | 281 | 375 | 80 | 64 |
| Cash benefit | 20 | 8 | 14 | 29 | -^a^ |
| Integration benefit | 39 | 48 | 49 | 5 | 14 |
| Unemployment support | 2768 | 2378 | 2123 | 933 | 751 |
| Pre-rehabilitation benefit | 1119 | 893 | 1093 | 433 | 482 |
| Rehabilitation benefit | 4634 | 2842 | 3248 | 1387 | 1000 |
| Early retirement pension | 4110 | 3080 | 3573 | 1874 | 2645 |
| Early retirement pension/social assistance | 403 | 336 | 532 | 181 | 256 |
| Resource course benefit | 3277 | 3135 | 2349 | 1463 | 1510 |
| Job assessment | 3480 | 2817 | 1703 | 1044 | 467 |
| Sickness benefit | 17478 | 12159 | 12452 | 4807 | 2638 |

^a^ not shown due for data privacy reasons

**Table S4.** Total effect of childhood adversity on social welfare dependence and 3-way decomposition, women only

|  | Total effect | Differential exposure | | Differential vulnerability | | Pure natural direct effect | |
| --- | --- | --- | --- | --- | --- | --- | --- |
|  | Additional cases per 1000 individuals | Additional cases per 1000 individuals | % of total effect | Additional cases per 1000 individuals | % of total effect | Additional cases per 1000 individuals | % of total effect |
| Low adversity | Ref. | Ref. | Ref. | Ref. | Ref. | Ref. | Ref. |
| Early material deprivation | 61 (58-65) | 9 (8-9) | 13.9 (12.5 – 15.3) | 3 (3-4) | 5.3 (4.5 – 6.1) | 50 (47-53) | 80.8 (79.2 – 82.4) |
| Persistent material deprivation | 133 (129-137) | 14 (13-14) | 10.1 (9.4 – 10.9) | 10 (9-11) | 7.2 (6.6 – 7.9) | 110 (106-114) | 82.6 (81.7 – 83.6) |
| Loss or threat of loss | 109 (103-114) | 21 (19-22) | 18.9 (17.5 – 20.3) | 9 (8-11) | 8.4 (7.0 – 9.8) | 79 (74-84) | 72.7 (70.8 – 74.6) |
| High adversity | 321 (311-331) | 48 (45-50) | 14.8 (14.0 – 15.7) | 30 (25-35) | 9.4 (7.8 – 10.9) | 243 (233 – 253) | 75.8 (74.1 – 77.5) |
| *Note. Discrepancies in summation to total effects are due to rounding. Estimates are adjusted for sex, birth year, parental country of origin, and maternal age at time of birth.* The estimates for differential exposure, differential susceptibility and the direct effect sum up to the total effect of each of the childhood adversity trajectories on social welfare dependence. | | | | | | | |

**Table S5.** Total effect of childhood adversity on social welfare dependence and 3-way decomposition, men only

|  | Total effect | Differential exposure | | Differential vulnerability | | Pure natural direct effect | |
| --- | --- | --- | --- | --- | --- | --- | --- |
|  | Additional cases per 1000 individuals | Additional cases per 1000 individuals | % of total effect | Additional cases per 1000 individuals | % of total effect | Additional cases per 1000 individuals | % of total effect |
| Low adversity | Ref. | Ref. | Ref. | Ref. | Ref. | Ref. | Ref. |
| Early material deprivation | 48 (45-50) | 7 (6-7) | 14.2 (12.6 – 15.8) | 2 (2-3) | 5.2 (4.2 – 6.2) | 38 (36-41) | 80.6 (78.8 – 82.4) |
| Persistent material deprivation | 111 (107-115) | 13 (12-14) | 11.4 (10.6 – 12.2) | 9 (8-10) | 7.8 (7.1 – 8.6) | 71 (66-75) | 72.3 (70.3 – 74.3) |
| Loss or threat of loss | 98 (93-103) | 18 (17-20) | 18.7 (17.3 – 20.2) | 9 (7-10) | 9.0 (7.5 – 10.4) | 71 (66-75) | 72.3 (70.3 – 74.3) |
| High adversity | 317 (308-326) | 49 (47-52) | 15.6 (14.7 – 16.4) | 32 (27-37) | 10.0 (8.5 – 11.5) | 236 (227 – 246) | 74.4 (72.8 – 76.1) |
| *Note. Discrepancies in summation to total effects are due to rounding. Estimates are adjusted for sex, birth year, parental country of origin, and maternal age at time of birth.* The estimates for differential exposure, differential susceptibility and the direct effect sum up to the total effect of each of the childhood adversity trajectories on social welfare dependence. | | | | | | | |

**Table S6.** Total effect of childhood adversity on social welfare dependence and 3-way decomposition, adjusted for parental education

|  | Total effect | Differential exposure | | Differential vulnerability | | Pure natural direct effect | |
| --- | --- | --- | --- | --- | --- | --- | --- |
|  | Additional cases per 1000 individuals | Additional cases per 1000 individuals | % of total effect | Additional cases per 1000 individuals | % of total effect | Additional cases per 1000 individuals | % of total effect |
| Low adversity | Ref. | Ref. | Ref. | Ref. | Ref. | Ref. | Ref. |
| Early material deprivation | 35 (33-37) | 1 (1-2) | 3.8 (2.3 – 5.4) | 4 (4-5) | 12.1 (11.3 – 12.9) | 29 (27-31) | 84.1 (82.5 – 85.7) |
| Persistent material deprivation | 89 (86 – 92) | 3 (2-4) | 3.5 (2.7 – 4.3) | 16 (15-16) | 17.5 (16.9 – 18.1) | 70 (68-73) | 78.9 (78.1 – 79.8) |
| Loss or threat of loss | 82 (78-86) | 13 (12-14) | 15.8 (14.6 – 17.0) | 11 (10-13) | 13.9 (12.8 – 15.0) | 58 (55 – 81) | 70.3 (68.7 – 71.8) |
| High adversity | 264 (258 – 271) | 35 (33-37) | 13.1 (12.4 – 13.9) | 54 (51 – 58) | 20.6 (19.4 – 21.7) | 175 (169-182) | 66.3 (65.1 – 67.5) |
| *Note. Discrepancies in summation to total effects are due to rounding. Estimates are adjusted for sex, birth year, parental country of origin, and maternal age at time of birth.* The estimates for differential exposure, differential susceptibility and the direct effect sum up to the total effect of each of the childhood adversity trajectories on social welfare dependence. | | | | | | | |

**Table S7.** Total effect of childhood adversity on social welfare dependence and 3-way decomposition, diagnoses only

|  | Total effect | Differential exposure | | Differential vulnerability | | Pure natural direct effect | |
| --- | --- | --- | --- | --- | --- | --- | --- |
|  | Additional cases per 1000 individuals | Additional cases per 1000 individuals | % of total effect | Additional cases per 1000 individuals | % of total effect | Additional cases per 1000 individuals | % of total effect |
| Low adversity | Ref. | Ref. | Ref. | Ref. | Ref. | Ref. | Ref. |
| Early material deprivation | 54 (52-56) | 5 (6-6) | 9.8 (8.9-10.7) | 2 (2-2) | 4.0 (3.4 – 4.6) | 46 (44-48) | 86.2 (85.2 – 87.3) |
| Persistent material deprivation | 121 (119 – 124) | 10 (9-10) | 8.1 (7.6 – 8.6) | 7 (7-8) | 5.9 (5.4 – 6.4) | 104 (102 – 107) | 86.0 (85.4 – 86.6) |
| Loss or threat of loss | 103 (99 – 106) | 16 (15-17) | 15.3 (14.4 – 16.3) | 7 (6-8) | 6.7 (5.7 – 7.6) | 80 (77 – 83) | 78.0 (76.7 – 79.3) |
| High adversity | 319 (312 – 325) | 41 (39 – 43) | 12.8 (12.2 – 13.3) | 20 (17 – 23) | 6.2 (5.3 – 7.2) | 258 (251 – 256) | 81.0 (80.0 – 82.1) |
| *Note. Discrepancies in summation to total effects are due to rounding. Estimates are adjusted for sex, birth year, parental country of origin, and maternal age at time of birth.* The estimates for differential exposure, differential susceptibility and the direct effect sum up to the total effect of each of the childhood adversity trajectories on social welfare dependence. | | | | | | | |

**References cited in the supplementary materials**

Nagin, D. S., Jones, B. L., Lima Passos, V., & Tremblay, R. E. (2018). Group-based multi- trajectory modeling. *Statistical Methods in Medical Research*, *27*(7), 2015- 2023. https://doi.org/10.1177/0962280216673085
